# Deep Learning for Human Disease Detection, Subtype Classification, and Treatment Response Prediction Using Epigenomic Data

**DOI:** 10.3390/biomedicines9111733

**Published:** 2021-11-20

**Authors:** Thi Mai Nguyen, Nackhyoung Kim, Da Hae Kim, Hoang Long Le, Md Jalil Piran, Soo-Jong Um, Jin Hee Kim

**Affiliations:** 1 Department of Integrative Bioscience & Biotechnology, Sejong University, 209 Neungdong-ro, Gwangjin-gu, Seoul 05006, Korea; mainguyen@sju.ac.kr (T.M.N.); nhkim@sejong.ac.kr (N.K.); dahae0218@sju.ac.kr (D.H.K.); umsj@sejong.ac.kr (S.-J.U.); 2 Department of Computer Science & Engineering, Sejong University, 209 Neungdong-ro, Gwangjin-gu, Seoul 05006, Korea; lehoanglong95@sju.ac.kr (H.L.L.); piran@sejong.ac.kr (M.J.P.)

**Keywords:** deep learning, epigenomics, disease detection, subtype classification, treatment response prediction, systematic review

## Abstract

Deep learning (DL) is a distinct class of machine learning that has achieved first-class performance in many fields of study. For epigenomics, the application of DL to assist physicians and scientists in human disease-relevant prediction tasks has been relatively unexplored until very recently. In this article, we critically review published studies that employed DL models to predict disease detection, subtype classification, and treatment responses, using epigenomic data. A comprehensive search on PubMed, Scopus, Web of Science, Google Scholar, and arXiv.org was performed following the Preferred Reporting Items for Systematic Reviews and Meta-Analyses guidelines. Among 1140 initially identified publications, we included 22 articles in our review. DNA methylation and RNA-sequencing data are most frequently used to train the predictive models. The reviewed models achieved a high accuracy ranged from 88.3% to 100.0% for disease detection tasks, from 69.5% to 97.8% for subtype classification tasks, and from 80.0% to 93.0% for treatment response prediction tasks. We generated a workflow to develop a predictive model that encompasses all steps from first defining human disease-related tasks to finally evaluating model performance. DL holds promise for transforming epigenomic big data into valuable knowledge that will enhance the development of translational epigenomics.

## 1. Introduction

Deep learning (DL) is a neural-network-based method that has multiple hidden layers [1] and is considered among the best paradigms of machine learning (ML) approaches for classification and regression [2]. DL has achieved great successes in handling the extensive heave of high dimensional and complex structured data of various fields of studies [3]. Because of its outstanding ability to solve tasks with higher accuracy than conventional methods, in the last decade, DL has emerged an important role in bioinformatics and systems biology to gain insights from an exponentially increasing amount of omics data [4].

Epigenetics was first introduced by Conrad Waddington in 1942 and has been widely accepted as “the study of changes in gene function that are mitotically and/or meiotically heritable and that do not entail a change in DNA sequence” [5], since then it has been considered as a novel approach to manage many complex diseases [6]. The epigenomic status of a cell or a tissue depends on a wide range of events such as DNA and histone modification, which are influent by environmental factors [7]. A comprehensive genome-wide catalog of epigenetic control elements and how this could be changed in different cell states can provide critical insights into the relationships among environmental exposure, genotype, and phenotype [6]. Existing evidence highlighted an important role of epigenetic biomarkers in a wide range of human diseases in terms of early detection, subtype classification, prognosis, and predicting response to therapy [8,9,10]. For this reason, translational epigenomics that ultimately seeks to leverage associations between epigenomic marks and clinical outcomes has received great concern in recent years [11].

The dramatic development of epigenomics poses challenges for traditional analysis methods in solving human diseases-related classification and regression tasks due to the large volumes of high-dimensional and high-throughput data. To overcome this issue, DL has been applied to take advantages of epigenomic data to assist medical professionals and researchers in improving understanding of human diseases. Although there have been a number of review papers regarding DL and epigenomics, only a limited number of review papers mentioned applicability of DL and epigenomics to clinical practices. In the last five years, ten comprehensive review articles have been published to shed the light on applications of DL to epigenomics [3,4,12,13,14,15,16,17,18,19] as presented in Table 1. Zhang et al. [3] and Min et al. [4] provided a useful guideline which allows researchers from various backgrounds to understand and utilize DL to solve omics-related problems, whereas Talukder et al. [12] attempted to unbox the black-box nature of DL, increasing the interpretability of DL in epigenomics. Nevertheless, these works focused on biological mechanisms and model structures rather than clinical outcomes of human diseases. In the same manner, previous reviews targeting cancer and rare diseases highlighted the promising ability of DL to elucidate the involvement of epigenomics in pathophysiology of human diseases, fostering novel diagnostic tools as well as therapeutic avenues [13,14,17,18,19]. Rauschert et al. [15] and Holder et al. [16] emphasized potential clinical applications of epigenetics and ML; however, the former only reviewed DNA methylation data, and the latter only provided a list of diseases or medical conditions without a comprehensive discussion. To conclude, there is a current gap of knowledge about the applicability of DL to solve human disease-related tasks using epigenomic data.

As a dramatically accelerating pace of development was witnessed in the field of DL and epigenomics for the last decade [3,4], we could foresee an exploration in integration of the two fields of studies in the near future to assist physicians in clinical practices. The primary reason for the delay of this trend could be a lack of communication between the two fields. In particular, epigenomics researchers who have a great deal of data get used to conventional statistical methods and mostly have no idea about how to make the best use of the data with DL, whereas DL researchers are in the opposite condition. We expect that our review is able to not only suggest fruitful collaborations between researchers in the two fields but also bridge the gap to a certain extent, and thus foster the applications of DL in translational epigenomics. In particular, our main perspectives include:

- Providing a thorough review about DL-based predictive models in epigenomics for disease detection, subtype classification, and treatment response prediction.
- Giving an insight into the main characteristics of the most common epigenomic data types and potential data sources, especially several publicly available databases, which could be used to develop the predictive models.
- Discussing data preprocessing flows, DL architectures, DL libraries, and model evaluation metrics that were feasible for epigenomics.
- Proposing current practical challenges and future trends of the development of epigenomic data-based DL techniques for translational medicine.

## 2. Materials and Methods

We conducted this review following the Preferred Reporting Items for Systematic Reviews and Meta-Analyses (PRISMA) guidelines [20].

### 2.1. Search Strategy

A comprehensive search strategy on the PubMed, Web of Science, and Scopus databases was developed to identify relevant articles published up to September 2021 without any data restrictions. The search queries combined key words relating to DL (e.g., common neural network architectures such as multi-layer perceptron, convolutional neural network, recurrent neural network, and autoencoder) and epigenomic data (i.e., DNA methylation, histone modification, and non-coding RNA). To identify additional relevant studies, we also performed a manual search on Google Scholar and arVix.org as well as checked the bibliography of the selected studies and key reviews. 

### 2.2. Study Selection and Eligibility Criteria

After importing initially identified articles to EndNote X9, we removed duplicates and then screened titles, abstracts, and full texts based on eligibility criteria as follows:

(1) DL models or predictive models that utilized DL as a component to solve human diseases-related tasks;
(2) Prediction tasks directly targeted clinical outcomes of human diseases (i.e., disease detection, subtype classification, prognosis, and treatment response prediction). We excluded articles that addressed biological mechanisms of diseases such as genes and gene sets prediction, characterization of chromatin states, and miRNA-disease associations.
(3) We focused on prediction applicability of epigenomic data including DNA methylation, histone modification, and non-coding RNA. Models using other omics data such as genomics, proteomics, transcriptomics, or multi-omics data were deleted;
(4) Only original works were included. Reviews, commentaries, and editorials were excluded;
(5) Publications with unavailable full texts were discarded.

For works that were improved and published more than once, we selected the latest publication only. Any disagreements among authors were solved by discussion until a consensus was reached. 

### 2.3. Data Extraction

We qualitatively synthesized the following data extracted from the included studies: names of the first author, years of publication, countries, target diseases, prediction tasks, types of data, data sources, data preprocessing methods, network architectures, validation schemes, and model performance. 

## 3. Results

### 3.1. Selection Results

**Figure 1:**
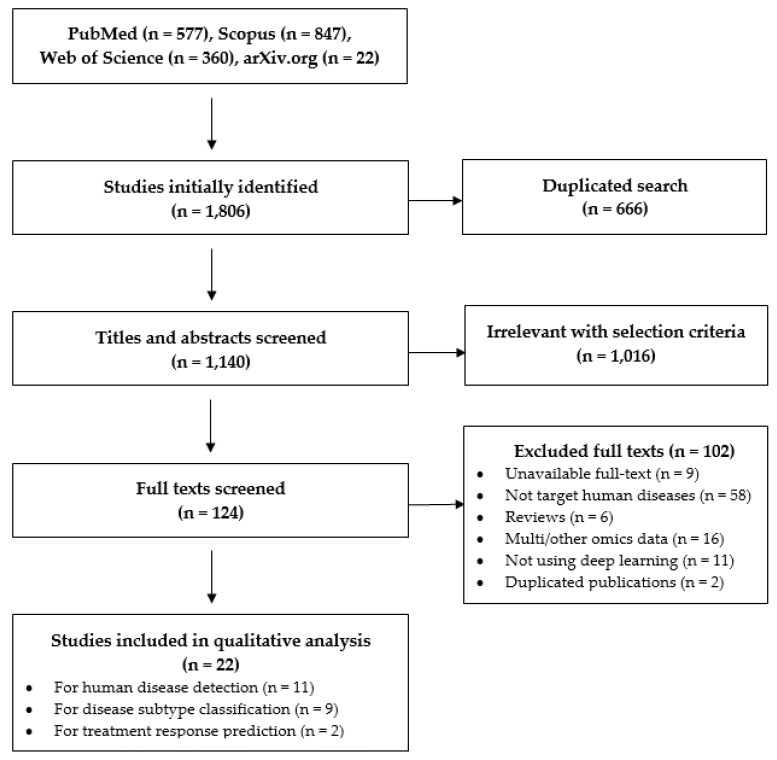
PRISMA flow for study selection.

The flow for study selection is presented in Figure 1. A total of 1806 studies were initially identified. After removing 666 duplicates, at the title and abstract screening step, we excluded 1016 articles that did not comply with the eligibility criteria. Full texts of 124 articles were extracted and screened for further detail. We eventually included 22 studies in our review.

### 3.2. An Overview of DL in Translational Epigenomics

Applications of DL to assist physicians and scientists in clinical settings using epigenomic data have been relatively unexplored until very recently. Except for one paper published in 2016, 21 out of 22 papers reviewed were published in the last 5 years and a majority of the models were developed by USA and China research teams [21,22,23,24,25,26,27,28,29,30,31,32,33,34,35,36,37,38,39,40,41,42]. This suggests a novel field of study that gains an increasing interest. Among human disease-related tasks, disease detection, subtype classification, and treatment response prediction received great concerns. Existing evidence indicated that DL models in epigenomics for solving the human disease-related tasks outperformed [23,24,29,41] or at least competitive to traditional ML models [37]. Some predictive models in previous studies utilized DL as a powerful component of a multi-step process [28,29,30,31,38,39]. About the epigenomic data type, DNA methylation and RNA-sequencing (RNA-seq) data are most frequently used. Various network architectures were employed such as multi-layer perceptron, autoencoder and its variants, convolutional neural network, and deep belief network. Reviewed models yielded high accuracy ranged from 88.3% to 100.0% for disease detection tasks [23,24,28,29,30,31], from 69.5% to 97.8% for subtype classification tasks [32,33,35,36,37,38,40], and from 80.0% to 93.0% for treatment response prediction tasks [41,42].

#### 3.2.1. DL in Epigenomics for Disease Detection

Until now, the predictive models using epigenomic data for disease detection primarily aim to differentiate subjects with health problems from healthy controls. Table 2 summarizes the main characteristics of DL in epigenomics for human disease detection.

Afshar et al. [21] and Alizadeh [22] proposed prediction toolboxes for colorectal and pancreatic cancer diagnosis, respectively, using miRNA expression profiles. To select the most important features for artificial neural networks (ANNs), Afshar et al. [21] calculated miRNA scores by artificial neural network units [43], while Alizadeh [22] performed particle swarm optimization (PSO). The two models achieved high performance, suggesting that miRNAs can be used as a sensitive and specific diagnostic marker.

Bahado-Singh et al. [23,24] recently proved that deep neural networks (DNNs) accurately predicted pediatric coarctation and concussion using DNA methylation data obtained from blood samples. These models outperformed five other frequently used ML approaches including random forest (RF), support vector machine (SVM), linear discriminant analysis, prediction analysis for microarrays, and generalized linear model. Interestingly, DL models using a combination of epigenomic and clinical markers yielded higher predictive accuracy.

Amor et al. [25] and Si et al. [29] developed two-stage models to identify cancer samples among normal samples using DNA methylation data. In both models, the dimensionality reduction was performed using autoencoder (AE) structures which allowed to extract features automatically. Si et al. [29] grouped extracted the features into cancer or non-cancer using k-means, Gaussian mixture method, and self-organizing map (SOM), while del Amor et al. [25] proposed a novel approach called deep embedded refined clustering (DERC) that trained end-to-end optimizing the dimensionality reduction and the unsupervised classification in the same step. These studies [25,29] found the followings:

- DNN-based extracted features were more effective for clustering analysis than those extracted from the principal component analysis (PCA) and non-negative matrix factorization (NMF),
- DNN and SOM outperformed previous probabilistic mixture methods [44,45,46],
- DERC achieved higher accuracy in breast cancer classification in comparison with other models under the same conditions.

Duan et al. [26] indicated that using the relative telomere length along with three gene promoter methylation levels, a back-propagation neural network predicted lung cancer with an accuracy higher than that of the Fisher discrimination model. The statistical analyses also strengthened associations between the four biomarkers and lung cancer.

Elias et al. [27] combined sequencing of circulating miRNA with a neural network for diagnosis of epithelial ovarian cancer. The model showed several advantages over CA125, a traditional diagnosis biomarker. In addition to outstanding performance in the prediction regardless of patient age, histology, or stage, biologic relevance of the model was tested, showing an intra-tumoral concentration of relevant miRNA.

Liu et al. [28] utilized the “moderated t-statistics” method [47] to discover 2000 CpG markers and 2000 promoter markers with the most differential methylation-related expression before employing two ML strategies, least absolute shrinkage and selection operator and RF, to identify final markers for each type. Two groups of methylation markers were then separately used as input data for two multi-layer feedforward neural networks. Prediction results show that cancer samples can be accurately distinguished from normal samples by both types of markers. This also suggests that the studied sets of methylation markers might be used for efficient and precise liquid biopsy of pan-cancers.

Xia et al. [30] proposed a convolutional neural network (CNN) based multi-model ensemble method using DNA methylation data to predict lung adenocarcinoma, liver hepatocellular carcinoma, and kidney clear cell carcinoma. Due to a small dataset scale and high-dimensional samples, a *t*-test was first applied to select significantly different methylation points. The selected features were subsequently fed into the first stage classification of five classical ML classifiers including Naïve Bayesian Classifier, k-Nearest Neighbor, Decision Tree, RF, and Gradient Boosting Decision Tree. Because no classifier outperformed the others in all the aspects, a CNN consisting of two convolution layers, a max-pooling layer, and a fully-connected layer was constructed to stack the prediction results of the multiple methods in the next step. The experiment results indicate that the proposed method is capable of uncovering the intricate relationship among the classifiers automatically and achieve superior performances.

Zhang et al. [31] introduced an attention-based DL method to classify schizophrenia patients from healthy controls. Similar to the model proposed by Si et al. [29], DNA methylation data were processed through a three-step flow that contains (1) feature subset selection by an attention-based fully-connected network which is able to learn the most important part of input data, (2) dimensionality reduction using deep autoencoder (DAE), and (3) schizophrenia detection using linear SVM. Good performance of the proposed method suggests a potential for schizophrenia classification on a real-world DNA methylation dataset.

LUAD, lung adenocarcinoma; LIHC, liver hepatocellular carcinoma; KIRC, kidney clear cell carcinoma; miRNA, microRNA; DNAm, DNA methylation; CS; cancer samples; NS, normal samples; TCGA, The Cancer Genome Atlas; GEO, Gene Expression Omnibus; CV, cross-validation; NN, neural network; ANN, artificial neural network; PSO, particle Swarm Optimization; DNN, deep neural network; VAE, variational autoencoder; MLP, multi-layer perception; SOM, self-organizing map; CNN, convolutional neural network; FC, fully-connected; DAE, deep autoencoder; SVM, support vector machine; AUC, area under the receiver operator characteristics curve; –, not available.

#### 3.2.2. DL in Epigenomics for Disease Subtype Classification

A majority of the predictive models for subtype classification have dealt with cancer. This is partly due to the public availability of large datasets such as The Cancer Genome Atlas (TCGA) and the Gene Expression Omnibus (GEO). Several research groups have investigated the use of variational autoencoders (VAEs) for unsupervised feature learning and dimensionality reduction as the first step in the subtype classification workflow. Table 3 presents applications of DL in epigenomics for disease subtype classification.

Al Mamun et al. [32] employed four DNNs (multi-layer perceptron (MLP), long short-term memory, CNN, and DAE) to explore the capability of long non-coding RNA (lncRNA) in classifying eight cancer types. CNN achieved the highest performance, while MLP achieved the poorest performance. In general, good classification results obtained from all models suggest that lncRNA expression is a significant feature to differentiate multiple types of cancer.

Deep2Met model [33] received preprocessed DNA methylation beta-values as input for a CNN consisting of five layers to predict whether cancer metastasized or not in a patient with colorectal cancer. The proposed model achieved the area under the precision-recall curve (AUPR), which was critical to estimate performance based on data with imbalanced classes, of 96.99%, as well as high values of sensitivity, specificity, accuracy, precision, and F-score. The results showed a promise for Deep2Met to diagnose colorectal cancer based on the methylation profiles of individual patients.

A class-incremental learning approach called Deep Generative Feature Reply was proposed for cancer classification tasks with superior accuracy [34]. The model is composed of an incremental feature selection for selecting the most significant CpG sites and a scholar network in which a VAE acted as a generator for generating pseudo data without accessing past samples and a neural network classifier acted as a predictor for cancer types.

Laplante et al. [35] developed a DNN classifier with the first layer consisting of 1046 input neurons for each stem-loop miRNA count and the final layer consisting of 27 neurons for each different type of cancer. Tumors in 20 anatomical sites were classified with 96.88% of accuracy, demonstrating the potential of miRNA data for an accurate cancer localization.

MethylNet [36] is a DNA methylation-based DL method that is capable of automatically constructing embeddings, making predictions, generating new data, and uncovering unknown disease heterogeneity. With regard to its structure, first, VAE was used to pre-train the DL model, which was to extract biologically meaningful features for clustering in the unsupervised setting. Second, prediction layers were included to fine-tune the encoder for tasks of multi-output regression and classification. Third, hyperparameter scans for the feature extraction network and the prediction layers were performed to optimize the model parameters. Eventually, predictions from MethylNet can be interpreted with two approaches including (1) Shapley Feature Attribution methods (SHAP) based on the contribution of the CpGs to each prediction and (2) comparing learned clusters of embedded methylation samples with corresponding subtypes for biological plausibility.

Smolander et al. [37] are pioneers in using non-coding RNAs beyond miRNAs for the classification of lung cancer patients with a deep belief network (DBN) and three state-of-the-art ML methods. The DBN was developed following an unsupervised pre-training phase with a restricted Boltzmann machine (RBM) and a supervised fine-tuning phase using the stochastic gradient descent either in combination with the basic backpropagation algorithm or the resilient backpropagation algorithm. Three main findings of this study were; (1) a competitive performance of the DBN to other classifiers, (2) an outweighed performance of the non-coding RNAs over coding RNAs, and (3) a negative effect of feature selection on the classification performance.

Titus et al. [38] and Wang et al. [39] developed a similar pipeline for the classification of breast cancer and lung cancer, respectively, using DNA methylation data. In further details, after employing Tybalt, a VAE model, to learn latent features of input data, the authors conducted dimensionality reduction using the t-distributed stochastic neighbor embedding (t-SNE), and then trained logistic regression classifiers to classify samples into one of their subtypes. The two studies demonstrated that the VAE provided a promising avenue for subtypes identification in precision medical research when the volume of publicly available methylation data is growing dramatically.

Zheng et al. [40] introduced a DNN-based classifier for cancer origin prediction using DNA methylation data. Performance of the proposed model was evaluated using four strategies including (1) 10-fold cross-validation, (2) hold-out testing data of 1468 patients, (3) 143 metastasized cancer patients with 12 origins, and (4) an independent dataset of 581 samples with 10 origins. All experiment results consistently showed a higher performance than existing pathology and gene expression-based techniques (Table 3). In addition, the DNA methylation-based DNN classifier had not only advantages of easy implementation in clinical settings but also potential for diagnosis of both unknown primary cancer and cancer cell types of circulating tumor cells. 

#### 3.2.3. DL in Epigenomics for Treatment Response Prediction

Treatment response prediction allows better personalized treatment, enhancing the development of precision medicine. DL model using epigenomic data has seldom been applied to predict treatment response, but interest in this approach has increased in recent years.

Chang et al. [41] investigated the pathogenic mechanism and biomarkers for hepatitis B virus drug development using a systematic approach based on big data mining and genome-wide RNA-seq data. As a part of this approach, a fully-connected neural network was employed to predict drug–target interactions. It outperformed three traditional ML methods including RF, k-Nearest Neighbor, and SVM with an accuracy of 92.6% (Table 4). In further detail, the network consisted of an input layer, four hidden layers, and an output layer with only one neuron which predicted the probability of a relationship between a drug and a target. This allowed the authors to focus on specific interactions, and thus to filter promising drugs based on pharmacological properties of the predicted drugs such as drug sensitivity, toxicity, and regulation ability.

Morrila et al. [42] developed a DNN-based classifier to predict responses to steroids, cyclosporine, or infliximab in patients with acute severe ulcerative colitis using miRNA expression profiles in colon tissues. Classification prediction of responders and non-responders to each treatment was achieved from nine miRNAs and five clinical factors that were routinely collected at the time of hospital admission with high accuracy as presented in Table 4.

### 3.3. An Insight into Epigenomic Data Used to Train Predictive Models for Human Diseases

#### 3.3.1. Types of Epigenomic Data

DNA methylation has been the most investigated epigenetic mechanism because of its roles on gene expression regulation including X-chromosome inactivation and allele-specific silencing of imprinted genes that are preferentially expressed from only one of the parental copies [48,49]. Integration between traditional biochemical methodologies and novel bioinformatic analysis methods such as DL extended our understanding for DNA methylation patterns in various types of human diseases such as cancer [25,26,28,29,30,33,34,36,39,40], concussion [21], schizophrenia [28], and cardiovascular diseases [20]. Profiling DNA methylation at a genome-wide level could be conducted using various types of sequencing technologies. Currently, the DNA methylation level is represented as beta-value, which is the ratio of methylation intensity to total methylation and unmethylation intensities [50]. The beta-value of each CpG locus is calculated using the following formula:

(1) $$\beta =\frac{max\left(I_{M},\;0\right)}{max\left(I_{M},\;0\right)+max\left(I_{U},\;0\right)+\alpha }$$

where $I_{M}$ and $I_{U}$ are the signal intensities representing methylation and unmethylation, respectively; α is an arbitrary offset that is usually set equal to 100 to deal with the case when fluorescent intensities are low [50]. According to this equation, the beta-value ranged between 0 and 1, corresponding with the completely unmethylated and methylated CpG site, respectively.

MicroRNAs (miRNAs) are endogenous small non-coding RNA molecules (21–25 nucleotides) that can regulate gene expression [51]. In further detail, when a miRNA interacts with its target messenger RNA (mRNA), usually in the 3′ untranslated region (UTR), the miRNA induces degradation or translational repression of the target RNAs depending on complete or incomplete complementarity, respectively. miRNAs are closely related to small interfering RNAs that have been shown to be involved in two profound epigenetic mechanisms, DNA methylation and histone modification [51]. Recent studies also found that miRNAs can be involved in establishing DNA methylation [52] and regulate chromatin structure by regulating key histone modifiers [51]. For these reasons, miRNAs can be considered to be important players in the epigenetic control of gene expression [53]. Reviewed studies proved their potential efficacy as non-invasive, specific, and sensitive biomarkers for disease diagnosis [21,22,27], subtype classification [35], and treatment response prediction [42].

As another class of non-coding RNAs, lncRNA is a single-stranded RNA with more than 200 nucleotides that is frequently transcribed by RNA polymerase II. Although lncRNAs biochemically resemble mRNAs, those molecules are not translated to protein, but coordinate and manage genetic regulatory outputs [54]. lncRNAs were regarded as junk in the past, but their molecular biological functions have received great concerns in recent years [55]. This is attributable to two primary reasons; first, misexpression of lncRNAs can cause changes in expression profiles of various target genes involved in human diseases, especially cancer [56]; second, lncRNAs are stable in body fluids due to their secondary structures [54]. With regard to the mechanism for how lncRNAs interfere with selective regions of the genome, there are three primary hypotheses about their functions including, (1) decoys that titrate away DNA-binding proteins (e.g., transcription factors), (2) scaffolds that bring two or more proteins into a complex or spatial proximity, and (3) guides that recruit proteins to DNA (e.g., through RNA–DNA interactions or RNA–DNA binding protein interactions) [54]. Taking the concept of lncRNAs as disease markers, Mamun et al. used lncRNA expression data for eight cancers (bladder urothelial carcinoma, cervical squamous cell carcinoma and endocervical adenocarcinoma, colon adenocarcinoma, head-neck squamous cell carcinoma, kidney renal papillary cell carcinoma, low-grade glioma, liver hepatocellular carcinoma, and lung adenocarcinoma) to develop DL models that were able to differentiate multiple cancer types with high accuracy, from 94% to 98% [32].

Because histone proteins are tightly wrapped by double-stranded DNA in the nucleus to compress DNA into chromatin, interaction between histones and DNA is crucial for gene activity [57]. In further detail, since DNA binds to an octamer structure of the histone complex, (H3, H4)_2_(H2A, H2B)_2_, histones may release or capture DNA to turn-on or turn-off gene expression, respectively. Furthermore, post-translational modification on histone proteins may change ionic charge around histone residues, affecting the histone–DNA interaction. Existing evidence proved that many human diseases are caused by mis-regulation of histone markers [58]. Taking advantage of this, coupled with the advancements in chromatin immunoprecipitation sequencing, several ML models were developed using histone modification data to discover biological mechanisms of diseases [13]. However, to the best of our knowledge, little has been known about applications of histone modification to predict clinical outcomes.

#### 3.3.2. Epigenomic Data Sources

Along with the rapid development of technologies profiling genome-wide sequencing, epigenomic data has exploded over the past decade. Table 5 presents data sources most frequently used to extract epigenomic data available for DL models.

Epigenomic data sources can be grouped into public datasets (i.e., publicly available and open access), which were contributed by specific studies, and private datasets (i.e., obtained from patients recruited in a specific study and for internal use only). Compared with private datasets which offer researchers full right to access data, open access databases have a limitation to access personal clinical data of interest which, in some cases, play an important role in understanding epigenomic status of patients. Additionally, private datasets are purpose-specific, whereas public datasets possibly lack data for several diseases. On the other hand, due to limited resources, private datasets could be deficient in diversity and number of patients compared to public databases. Because generalizing biological interpretation in human diseases is crucial within age groups, cultural, and racial variances, results from public datasets could have more reliable biological meaning than private databases.

DL researchers may collect epigenomic data from single or multiple data sources. The most two common public databases for epigenomic data are TCGA and GEO. The TCGA database was made by a joint effort of the National Cancer Institute and the National Human Genome Research Institute to generate comprehensive and multi-dimensional maps of genomic changes on more than 11,000 cancer cases from 33 different cancer types [59]. A vast amount of DNA methylation and RNA-seq data are accessible to researchers belonging to the cancer research community through the Genomic Data Commons data portal (https://portal.gdc.cancer.gov/ accessed on 27 October 2021). TCGA provides three levels of data that are defined in terms of processing level (raw, normalized, or integrated). Specifically, level 1 typically indicates raw and un-normalized data; level 2 typically indicates normalized and/or intermediately processed data; and level 3 typically indicates integrated, normalized, and/or segmented data. The results of integrative or pan-cancer analyses are sometimes referred as level 4. Of these, level 1 data account for the vast majority.

The GEO database was launched in 2000 by the National Center for Biotechnology Information (NCBI) as an international public repository for high-throughput genomic datasets [60]. It accepts both raw and processed data obtained using a wide range of technologies, including DNA microarrays, high-throughput nucleic acid sequencing, protein or tissue arrays, serial analysis of gene expression, and reverse transcription polymerase chain reaction. Although approximately 90% of the data in GEO are gene expression data, this database also provides comprehensive data sets for DNA methylation, RNA-seq, and other types of omics.

Other online data platforms provide a number of qualified genome-wide and clinical datasets. For instance, the current release of Epigenome-Wide Association Study (EWAS) Data Hub (https://bigd.big.ac.cn/ewas/datahub accessed on 27 October 2021) provides a collection of DNA methylation data from 75,344 samples, involving 67 diseases [61]. In addition, appearance of novel web tools for genome-wide research would induce qualified and user-friendly programs which might help extend researchers’ understanding about epigenomic mechanisms of human diseases. For example, SurvivalMeth links clinical data to DNA methylation of patients [62]. MethDB provides web analyzing tools for DNA methylation; however, the site was barely updated [63].

### 3.4. A Workflow for Developing a Predictive Model in Translational Epigenomics

**Figure 2:**
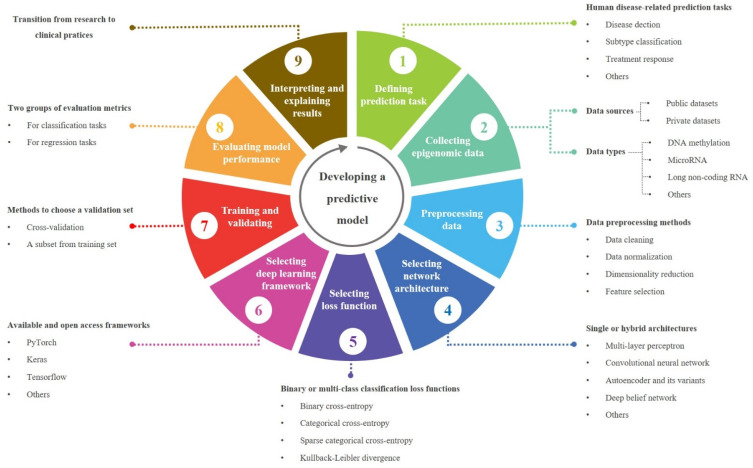
A workflow for developing a predictive model in translational epigenomics.

We summarized the reviewed models to generate a workflow for developing a predictive model that is able to solve human disease-related tasks using epigenomic data in Figure 2. In this section, we focused on preprocessing methods, network architectures, DL libraries, and evaluation metrics.

#### 3.4.1. Data Preprocessing

**Figure 3:**
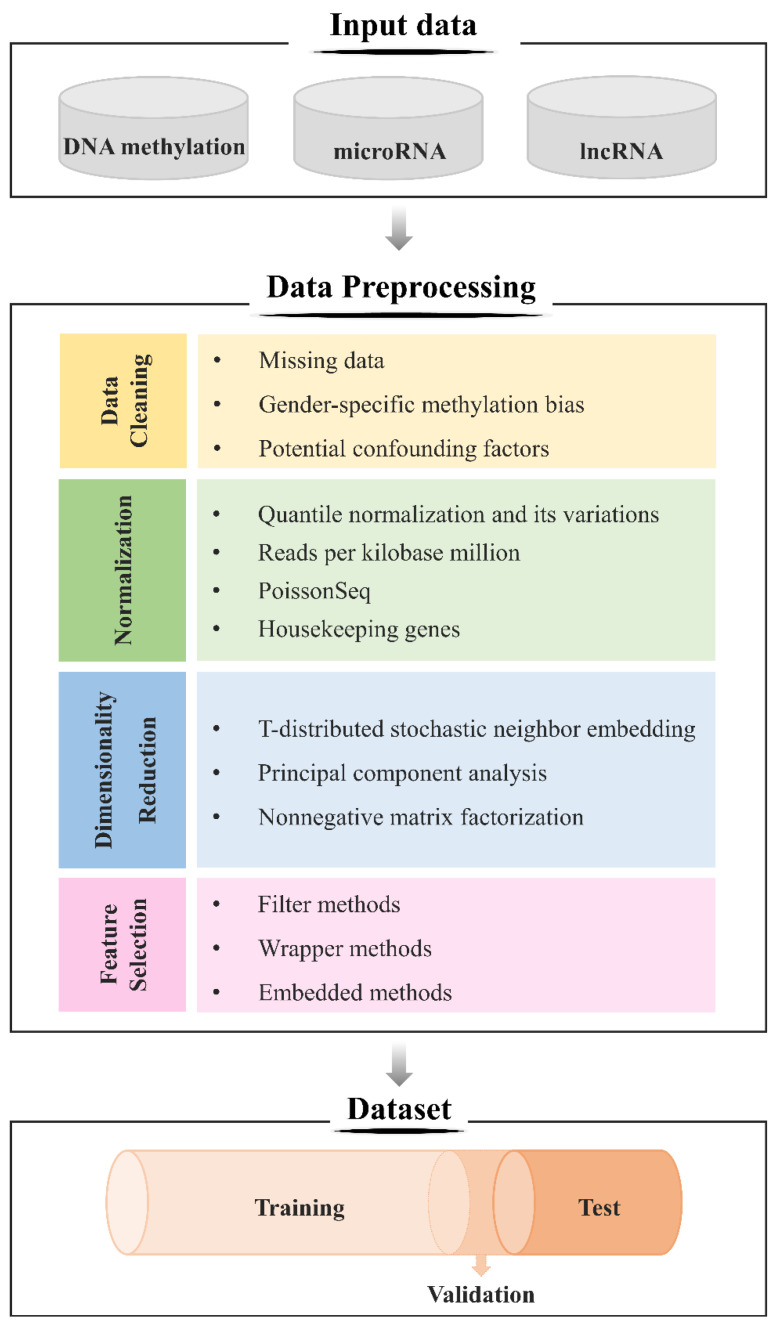
Data preprocessing flow for developing a predictive model in epigenomics.

Although DL models are capable of automatically learning the features of data, proper preprocessing of the data can greatly improve the accuracy and speed of the DL model. Data preprocessing includes several steps including importing data, summarizing and plotting row data, imputing missing values, normalizing and standardizing, handling outliers, analyzing data, and interpretation validating. The most commonly used preprocessing methods for epigenomic data include data cleaning, normalization, dimensionality reduction, and feature selection. A flow for raw data processing is presented in Figure 3.

Data cleaning improves the quality of data by detecting and removing errors and irregularities caused by inconsistencies or misspellings during data entry, missing information, and the integration of heterogeneous data sources [64]. In particular, with regard to the DNA methylation data, researchers should consider missing data, gender-specific methylation bias, and potential confounding factors. First, there are some solutions to handle missing values such as filtering, replacing missing values by zero, replacing by the mean or median value, and employing K-nearest neighbor imputation or expectation-maximization. For the private datasets, missing values are mostly caused by a low level of methylation (i.e., below the detection limit), and thus they are generally replaced with half of the minimum value in the original data [23]. For the public datasets, the CpG sites with missing values were deleted [28,31,33,39]. Second, to avoid potential gender-specific methylation bias related to significant CpG sites on sex chromosomes, CpG probes on the X and Y chromosomes were also removed [23,24,38,39,65]. Third, potential confounding factors were minimized by excluding the CpG probes which have known single nucleotide polymorphisms (SNPs) between 0 and 10 base pairs distance [23,24,38,39] because SNPs near or within the probe sequence may influence corresponding methylated probes [66]. In relation with miRNA stem-loop counts obtained from public databases, high correlations could be produced among some of the datasets. In such cases, the highly correlated datasets were grouped together based on anatomical site [35]. Furthermore, for the miRNA expression level, Afshar et al. [21] removed miRNAs with signal-to-noise ratio smaller than or equal to 2.5, whereas Elias et al. [27] selected a detection threshold at 10 tags per million read.

Normalization and standardization are employed to adjust the measurements in order to properly compare the samples. Data normalization involves the transformation of features into a common range for greater numeric feature values not to dominate the smaller numeric feature values, and thus minimizes the bias of these features [67]. There are two types of normalization for epigenomic data including, (1) between-array normalization removes technical artifacts which could be produced among the same samples on different arrays and (2) within-array normalization corrects for intensity-related biases which could be produced concentration-dependent [50]. To guarantee a correct normalization, researchers should consider assumptions that go along with the normalization methods. Among various methods for DNA methylation data, quantile normalization is one of the most commonly used techniques [26,68,69]. Following its popularity, many quantile normalization-based variations such as subset-quantile within array normalization, stratified quantile normalization, and beta-mixture quantile method have been developed; however, all these methods assume that global methylation does not vary between samples [70]. On the other hand, RNA-seq data are frequently normalized by library size (e.g., reads per kilobase million), by distribution (e.g., quantile normalization), by testing (e.g., PoissonSeq), or by controls (e.g., housekeeping genes) [71]. Alizadeh et al. [22] and Laplante et al. [35] utilized Min–Max Normalization, which transforms the minimum, maximum, and remaining values into 0, 1, and decimals between 0 and 1, respectively, to normalize the miRNA expression level.

The extremely high dimension of the epigenomic data yield many practical problems in training DL models. First, various conventional dimensionality reduction methods based on a Gaussian distribution assumption such as PCA [72] and NMF [73] cannot adapt to epigenomic data which follow a non-Gaussian distribution. Second, a combination of the high dimensionality and small sample sizes due to high cost and limitation of experiment environment to obtain epigenomic data raises a great concern about the curse of dimensionality [74] as well as overfitting problem [31], all of which can deteriorate the performance of a DL model. Dimensionality reduction is the transformation of high-dimensional data into low-dimensional data, which ideally correspond to the intrinsic dimensionality of the data. The t-SNE method, a nonlinear dimensionality reduction technique, is commonly used to compress features and visualize epigenomic data in two- or three-dimensional spaces using a scatter plot [75]. Unsupervised hierarchical clustering was then conducted on the t-SNE features for subtype classification [39].

Selecting a subset of features helps to decrease training time, increase model interpretability, and generalize performance on the test set. There are three categories of commonly used supervised feature selection methods including filter, wrapper, and embedded [31]. For example, *t*-test, Wilcoxon rank sum, and F-test with a threshold of *p*-value were applied to filter significant biomarkers [25,27,30,42]. Subsequently, the *p*-value was adjusted using the Benjamini–Hochberg correction for false discovery rate [23,24,28]. Si et al. [29] and Batbaatar et al. [34] selected features based on variance-based filtering techniques. Interestingly, an attention-based fully connected network for feature selection was proposed to generate a sparse representation of the input features [31].

In recent years, several DL models such as autoencoder and its variants have been employed for both dimensionality reduction and feature selection [25,29,31,36,38,39]. Several convenient high-throughput preprocessing workflows for epigenomic data were developed to simplify and accelerate data preparation for training DL models. For example, PyMethylProcess, a preprocessing pipeline for DNA methylation data built using Python version 3.6, allows users to control data quality (i.e., bead number, background correction, detection *p*-value, and outlier), remove non-autosomal and SNP sites, normalize and impute data, and eventually select appropriate features [74].

Following the above-mentioned steps, the dataset is randomly split into two groups including a training set and a test set which, in most cases, contains 80% and 20% of the study subjects, respectively. There are two common ways to select a validation set from the training set to tune hyperparameters and select a model with the highest performance. The first method is extracting 20% of data through random selection of the training set. The second method is using cross-validation, a resampling technique that is the gold standard for error estimations to avoid bias [76]. In specific, the training set is divided into k-folds whereby (k-1) folds are used for training and one fold is used for testing.

#### 3.4.2. Loss Function

The primary purpose of training a DL model is to minimize the loss function (i.e., the difference between the predicted value and the actual value) [77]. This task is done using an algorithm, e.g., backpropagation, which propagates the prediction error of a neural network backward, from the output layer to the input layer, so that weights of each connection can be adjusted [77,78]. There are heaps of loss functions for regression and classification tasks. As all existing DL models for translational epigenomics dealt with the latter [21,22,23,24,25,26,27,28,29,30,31,32,33,34,35,36,37,38,39,40,41,42], this section covers the most commonly used binary and multi-class classification loss functions.

A prime representative of the classification loss functions is cross-entropy loss function which was originated from the idea of entropy from information theory (i.e., the number of bits required to transmit a randomly elected event from a probability distribution). It refers to a measure of the difference between two probability distributions for a given random variable or set of events and can be calculated as follows [79].

(2) $$Loss=-\underset{x\epsilon classes}{\sum }P\left(x\right)\cdot \log\left(Q\left(x\right)\right),$$

where *P(x)* is the true probability distribution, *Q(x)* is the predicted probability distribution. 

Binary cross-entropy separately deals with each individual output whose value is either 0 or 1, while categorical cross-entropy is designed for multi-class classification with one-hot vector ground truth, meaning that only target class receives value 1 and all remaining classes receive value 0. For instance, the output layer of the network proposed by Laplante et al. [35] was composed of 27 neurons corresponding with 27 types of cancer. A categorical cross-entropy loss function, coupled with sigmoid activation function which generated the probability of a specific class, was employed to train the model.

As a side note, in the case that the classes are mutually exclusive and integer encoded, a sparse categorical cross-entropy can be considered to be more beneficial than categorical cross-entropy in terms of training time, memory, and computation.

In spite of being less common than the aforementioned multi-class classification loss functions, Kullback–Leibler (KL) divergence loss, which is also known as relative entropy, was also utilized in training DL models in epigenomics [25].

#### 3.4.3. Network Architectures

**Figure 4:**
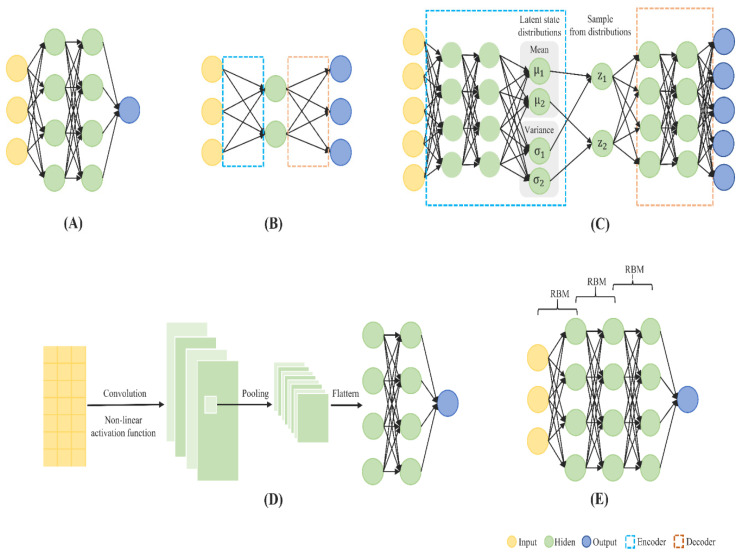
DL architectures that have been applied in epigenomics to solve some human diseases-related prediction tasks. (**A**) Multi-layer perceptron, (**B**) Autoencoder, (**C**) Variational autoencoder, (**D**) Convolutional neural network, (**E**) Deep belief network.

An ANN is composed of nodes that are grouped into connected layers and take the output from the last layer’s neurons through weighted connections [80]. The weight matrix is optimized during the training procedure to minimize the difference between the predicted values and the ground truths [81]. A DNN is basically an ANN of multiple non-linear layers that is typically composed of an input layer, multiple hidden layers, and an output layer. Each layer contains a number of computational units carrying out the transformation of the data received from the previous layer, then passing the results to the next layer. There are a wide variety of DNNs that have been applied in epigenomics to solve human disease-related tasks, some of which are illustrated in Figure 4.

An MLP is also known as a multi-layer neural network that fully connects multiple layers in a directed graph, meaning that the signal path through the nodes is always feed-forward. Training an MLP involves constantly adjusting for weights of the network using a backpropagation learning algorithm as a supervised learning technique so that an optimized network can be established between the input and output layers [82]. Similar to other pure supervised learning method, MLP requires a large amount of labelled data for training. MLP is widely used when features are not related in time or space in epigenomic studies. Mamun et al. [32] and Zheng et al. [40] employed MLP for classifying multiple cancer types using lncRNA and DNA methylation data, respectively.

An AE is a type of ANN that is typically designed for dimensionality reduction and feature representation learning in an unsupervised manner before using other ML or DL methods for prediction [83]. A basic AE is composed of encoding and decoding stages (also known as encoder and decoder, respectively), which are separated by the central bottleneck. While the encoder produces a code which is a low-dimensional latent representation of the original input data, the decoder attempts to reconstruct the input from the code [84]. For example, Zhang et al. applied a DAE, which is formed by stacking several AEs to reduce the dimension of the features, then passed the vector output to a linear SVM for disease classification [31].

A VAEs is an unsupervised learning model, which learns latent representations of input data through data compression and nonlinear activation functions [85]. VAE models are stochastic and learn the distribution of explanatory features over samples during training. Tybalt, a commonly used VAE model trained on the TCGA data by Way et al. [86], is capable of generating meaningful latent spaces for image and text data. It consists of an Adam optimizer, Rectified Linear Unit (ReLU), and batch normalization in the encoder, and a sigmoid activation in the decoder.

A CNN is also a feedforward neural network that uses convolution in place of general matrix multiplication in at least one of layers [77]. A CNN typically consists of three components including, (1) parallel convolution operations to produce a set of linear activations, (2) a nonlinear activation function, and (3) a pooling function to modify the output of the layer [87]. In further detail, convolution is central to any CNN and involves combining an input matrix with a kernel to produce an output feature map. Three most important attributes of convolution include sparse interactions (i.e., making the kernel smaller than the input), parameter sharing (i.e., using the same parameter for more than one function in a model), and equivariant representations (i.e., if the input changes, the output changes in the same way). The pooling function replaces the output of a net at a certain location with a summary statistic of the nearby outputs, and thus, reduces the training parameters as well as the degree of overfitting (i.e., the condition that a model fails to fit data properly or predict future observations reliably because of excessive fitting of the training data). For example, max pooling technique reports the maximum value within a rectangular cluster of neurons in a feature map.

DBN that typically consists of several RBM layers for unsupervised pre-training and one backpropagation layer for tuning parameters using labeled data (i.e., supervised fine-tuning). Smolander et al. [37] compared the classification performance of different versions of DBN with SVMs, decision trees, and RF for lung cancer using RNA-seq data and found that DBN performed at least competitively to these ML classifiers.

A recurrent neural network (RNN) is a distinct class of ANNs characterized by the existence of cycles in the networks that is typically due to edges that connect adjacent time steps (recurrent edges). Nodes with incoming recurrent connections can receive as input not only the current data point but also the values of hidden units from previous time steps. This makes RNNs suitable to model data that are sequential in nature, such as natural language or time series. For this reason, RNN has not been widely employed on epigenomic data.

#### 3.4.4. DL Libraries

Back to 1986 when DL was first being introduced, building a DL model was difficult due to objective and subjective reasons including unabundant data, inadequate hardware infrastructure, and numerous algorithmic details of a neural network that needed to be considered [88]. The explosion of big data and advancement of hardware such as graphics processing unit (GPU) have fostered the application of DL in the last decades. To support researchers from various fields of study without a computing background to take advantage of DL, numerous open-source and freely available DL libraries that are capable of simplifying the process of developing a DL model have been created. Table 6 briefly describes several libraries for training the DL models that are widely adopted in epigenomics. They are diverse in terms of function, programming language, neural network type supported, and applicable operating system.

#### 3.4.5. Model Evaluation Metrics

Existing DL-based predictive models for human disease-related tasks including disease detection, subtype classification, and treatment response prediction primarily dealt with classification tasks whose outcome is basically a discrete variable [21,22,23,24,25,26,27,28,29,30,31,32,33,34,35,36,37,38,39,40,41,42]. Table 7 summarizes the main characteristics of common metrics used to evaluate the classification performance of a DL model.

## 4. Challenges and Future Research Directions

In recent years, following the great successes of DL in bioinformatics, numerous researchers have applied DL methods to epigenomics to solve problems related to human diseases. However, this research topic is still in an early stage with target diseases primarily focusing on cancer and prediction models mostly dealing with disease detection and subtype classification tasks. Further investigation on other chronic diseases and prediction tasks related with prognosis and treatment response should be taken into consideration.

One of the biggest challenges for developing a DL model in epigenomics is the limited and imbalanced data because sufficient and balanced data are required to achieve a well-performed model with a tremendous number of weight parameters [89]. This challenge can be alleviated by increasing the sample size. However, it does not hold in the epigenomic field because complex and expensive data acquisition processes cause difficulties in getting reliable and big data on demand. For this reason, potential alternative methods are divided into three main groups including (1) data preprocessing which typically provides a better dataset, (2) cost-sensitive learning which replaces the objective loss function based on data classes during training, and (3) algorithmic modification which accommodates the learning algorithm to increase their suitability [89]. Of these, the currently reviewed models for human diseases cover mostly applied feature selection, which is a data preprocessing method, to alleviate curse of dimensionality and overfitting problem, and thus to improve the performance.

In this review, we did not deal with multi-omics data because of challenges in combining heterogeneous data which were obtained using different processing methods. Existing evidence suggests that DL models combining data in various formats could hold great promises in predicting disease diagnosis, classification, and treatment outcomes. For example, DNA methylation, miRNA, and gene expression data can be used to predict paclitaxel response among patients with breast cancer [90], survival in liver cancer [91], and metastasis status of various types of cancers [92]. Park et al. [93], Hira et al. [94], and Baek et al. [95] found that prediction models for Alzheimer’s disease, ovarian cancer, and prognosis for different cancer types, respectively, using multi-omics data yielded higher accuracy compared with prediction models solely using DNA methylation data. These results were consistent with findings obtained from DL models using DNA methylation data with clinical data [23,24]. Nevertheless, it is worth noting that there may raise a question about the interpretation of the prediction results with regard to contribution of each type of data.

Although DL produces outstanding performance in predicting disease detection, subtype classification, and treatment response, little has been known about how prediction results are generated, raising a great concern about a lack of interpretability [4]. This black-box nature of DL limits its application to health-related problems because for clinical practices, understanding mechanisms of how to produce good prediction results is as important as producing them [96]. Furthermore, despite an extensive use of public epigenomic datasets, validating a published model in terms of reproducibility, replicability, and generalizability seems to be impossible due to a lack of code sharing. For these reasons, transition from research to clinical setting of the reviewed models requires careful consideration and adequate descriptions for validation. Recently, great effort has been made to transform DL from a black-box into a white-box using visualization approaches [97]. Zheng et al. [40] suggested that a hybrid approach combining existing pathological examinations with epigenomic data-based prediction may offer both high interpretability and high prediction power. In addition, discovering biomarkers that are able to explain pathogenic mechanism for drug response may partially contribute to the applicability of DL model to pharmacology, as described in the work of Chang et al. [41]. To a certain degree, MethylNet is capable of interpreting prediction results using two approaches including (1) SHAP and (2) comparing learned clusters of embedded methylation samples with corresponding subtypes [36]. Even so, the interpretation of DL models towards applicability to medicine is still far from the goal.

Selecting an appropriate DL architecture that fits input data characteristics and research objectives is of primary importance to obtain robust and reliable results. However, up to now, a detailed methodology for model selection remains as a practical issue that requires further investigation. This issue may be partially addressed by comparing performance of several published models with diverse types of architectures in respect of solving similar tasks. More importantly, even when a DL architecture is selected, there are a great number of hyperparameters needed to be set beforehand. However, tuning hyperparameters is mostly subjective and rarely thorough, highlighting an important role of DL experts. This inhibits researchers in the field of epigenomics who may only have basic computational expertise to optimize DL models. Therefore, automatically optimizing hyperparameters is attracting great attention [98]. Furthermore, although having numerous learning parameters is a great advantage of DL to improve the performance, it poses a risk for overfitting, especially when the number of parameters is large and the dataset is small [99]. Another issue with regard to parameters and hyperparameters that should be taken into consideration is that a drastic increase in training and inference times of DL, compared with those of traditional ML models, emphasizes an urgent need for DL acceleration. Fortunately, advanced developments of DL algorithms and GPU-based implementation have enabled DL to run in a much shorter time. However, as epigenomic data is growing at a fast pace, novel DL frameworks should be further investigated to improve training efficiency and prediction accuracy.

## 5. Conclusions

In this article, we systematically reviewed 22 DL-based predictive models for human disease detection, subtype classification, and treatment response prediction using epigenomic data. Our summaries and comparisons in terms of prediction tasks, data types, data sources, neural network architectures, model structures, and prediction performance could be useful for interested researchers to develop and/or improve their models properly. Such pioneer models outperform traditional ML models, holding a great potential for their applicability in the clinical settings in the future. However, actual applications are still far from the reality due to a lack of validation replicated and insufficient interpretability. There is still room for improving predictive models by increasing the interpretability of DL and developing a detailed methodology for model selection. This review may strengthen the bridge between DL and epigenomics, and thus foster the applications of DL in translational epigenomics in the near future.

## Figures and Tables

**Table 1:** Summary of previous reviews about DL and epigenomics.

Research	Title	Main Findings
Zhang et al. (2019) [3]	DL in Omics: A Survey and Guideline	The combination between DL and omics is a novel promising approach, thus requires further investigation. This survey summarizes several applications of DL in genomics, epigenomics, transcriptomics, and proteomics, then provided a guideline for this topic.
Min et al. (2017) [4]	DL in Bioinformatics	Network architectures that have been utilized in bioinformatics include DNNs (MLP, SAE or DBN), CNNs, RNNs, and emergent architectures (DST-NNs, MD-RNNs, and CAEs). Limitations of DL in omics studies are (1) limited and imbalanced data, (2) black-box problem, and (3) selection of DL architecture and hyperparameters.
Talukder et al. (2020) [12]	Interpretation of DL in Genomics and Epigenomics	Various studies about motif finding, epigenomics, chromatin interaction prediction, gene expression prediction as well as ncRNA identification and regulation utilized DL feature interpretation techniques. The most popular methods for CNN-based DNNs interpretation include (1) input modification methods, deconvolutional methods, and (3) input reconstruction methods, whereas RNN-based DNNs frequently used attention mechanism along with other interpretation methods.
Arslan et al. (2021) [13]	ML in Epigenomics: Insights into Cancer Biology and Medicine	The complexity, sparsity, high-dimensionality, and noise of epigenomic data pose great challenges for analysis. This review discusses our major ML categories including (1) dimensionality reduction, (2) unsupervised methods, (3) supervised methods, and (4) DL. Non-negative matrix factorization is a popular clustering and dimensionality reduction method in epigenomics.
Brasil et al. (2021) [14]	Artificial Intelligence in Epigenetic Studies: Shedding Light on Rare Diseases	Applications of ML in epigenomic data analysis contribute to the improvement of diagnosis rate, discovery of biomarkers, and development of potential therapy for rare diseases. Future studies should avoid misinterpretation of data. The small number of studies found suggests that this is a novel field of study open to expansion.
Rauschert et al. (2020) [15]	ML and Clinical Eepigenetics: A Review of Challenges for Diagnosis and Classification	This review provides an overview of epigenomics and promising applicability of ML in clinical practices. Several challenges remain to combine epigenetics with ML including (1) cross-jurisdiction collaboration is needed to generate huge datasets, (2) the number of variables is larger than that of samples, (3) non-linear associations in DNA methylation datasets, (4) epigenetic datasets should be publicly available, and (5) prediction bias. DL outperforms traditional ML in terms of classification tasks. However, DL should only be used as an assistive tool until what happens in the “black box” is defined.
Holder et al. (2017) [16]	ML for Epigenetics and Future Medical Applications	ML can be used in medical records, population-based epidemiology, and identification of molecular information to assist the diagnosis and treatment of a wide range of diseases. The authors propose a combination of active learning, imbalanced class learning, and DL as a promising direction toward future medical applications.
Fan et al. (2018) [17]	ML Methods in Precision Medicine Targeting Epigenetic Diseases	This review provides a workflow of ML in epigenetics research. Supervised learning methods are commonly used for prediction, whereas unsupervised learning methods are mostly used for data cleaning and feature extraction. Although ML has gained outstanding achievements in epigenetics studies related to precision medicine, clinical applications is still far from the goal.
Iestao et al. (2021) [18]	Role of Regulatory Non-Coding RNAs in Aggressive Thyroid Cancer: Prospective Applications of Neural Network Analysis	ncRNAs can be potentially used as biomarkers for diagnosis of thyroid cancer as well as prediction of tumor aggressiveness. This review suggests an approach using DNNs to predict ncRNA molecular for early detection and prognosis of thyroid cancer.
Jovčevska et al.(2020) [19]	Next Generation Sequencing and ML Technologies Are Painting the Epigenetic Portrait of Glioblastoma	Epigenetics in glioblastoma is a novel approach that holds potential for identification of clinical biomarkers for diagnosis or discovery of drug targets. Training ML and DL algorithms using next generation sequencing data can produce comparable and consistent diagnoses without human errors, but still need to be improved to adapt their results to the nature of the disease.

DL, deep learning; DNN, deep neural network; MLP, multi-layer perceptron; SAE, stacked auto-encoder; DBN, deep belief network; DST-NNs, deep spatio-temporal neural networks; MD-RNNs, multi-dimensional recurrent neural networks; CAEs, convolutional auto-encoders; CNN, convolutional neural network; RNN, recurrent neural network; ML, machine learning; ncRNA, non-coding RNA.

**Table 2:** Comparison of DL models for disease detection using epigenomic data.

Research	Country	Target Disease	Data Type	Epigenomic Data Source	Validation Scheme	Predictive Model	Evaluation Metrics					
							AUC	Sensi-tivity	Speci-ficity	Accu-Racy	Preci-Sion	F1-Score
Afshar et al. (2019) [21]	Iran	Colorectal cancer	miRNA	50 CS and 150 NS (GSE59856–GEO)	15% of the dataset	ANN	1.000	0.900	0.970	1.000	–	–
Alizadeh et al. (2020) [22]	Iran	Pancreatic cancer	miRNA	GSE113486; GSE59856; GSE85589; GSE106817; GSE112264; GSE124158 (GEO)	5-fold CV on training and testing sets	ANN + PSO	–	0.930	0.920	0.930	–	–
Amor et al. (2021) [25]	Spain	Breast cancer	DNAm	GSE32393; GSE57285; GSE50220 (GEO)	10% of the dataset	VAE	–	–	–	0.993	–	–
Bahado-Singh et al. (2020) [23]	USA	Coarctation of the aorta	DNAm	24 cases and 16 controls	10-fold CV on training set (80% of the dataset)	DNN	0.970	0.950	0.980	–	–	–
Bahado-Singh et al. (2020) [24]	USA	Concussion	DNAm	17 cases and 18 controls	10-fold CV on training set (80% of the dataset)	DNN	0.989	0.950	0.912	–	–	–
Duan et al. (2017) [26]	China	Lung cancer	DNAm	200 CS and 200 NS	–	Back-propagation NN	0.760	–	–	–	–	–
Elias et al. (2017) [27]	USA	Ovarian cancer	miRNA	179 human serum samples	51 independent clinical samples	MLP	0.900	–	1.000	–	0.913	–
Liu et al. (2019) [28]	China	Pan-cancer (27 types)	DNAm (CpG markers)	10,140 CS and 3386 NS (TCGA and GEO)	370/4840 CS, 201/1742 NS	Two multi-layer feedforward NNs	0.989	0.928	0.901	0.924	–	–
			DNAm (Promoter markers)				0.985	0.898	0.811	0.883	–	–
Si et al. (2016) [29]	China	Breast cancer	DNAm	113 CS and 23 NS (GSE32393–GEO)	–	Auto-encode DNN + SOM	–	–	–	0.971	–	–
Xia et al. (2019) [30]	China	LUAD	DNAm	460 CS and 32 NS (TCGA)	5-fold CV on the whole datasets	CNN based ensemble model	0.998	–	–	0.994	–	–
		LIHC		379 CS and 50 NS (TCGA)			0.994	–	–	0.988	–	–
		KIRC		320 CS and 160 NS (TCGA)			0.999	–	–	0.996	–	–
Zhang et al. (2020) [31]	China	Schizophrenia	DNAm	54 cases and 18 controls	10-fold CV on the whole dataset	Attention-based FC + DAE + SVM	–	0.998	0.988	0.991	–	–

DNAm, DNA methylation; ncRNA, non-coding RNA; miRNA, microRNA; TCGA, The Cancer Genome Atlas; GEO, Gene Expression Omnibus; CV, cross-validation; CNN, convolutional neural network; DAE, deep autoencoder; MLP, multilayer perceptron; LSTM, long short-term memory; ANN, artificial neural network; VAE, variational autoencoder; DBN, deep belief network; AUC, area under the receiver operator characteristics curve; –, not available.

**Table 3:** Comparison of DL models for disease subtype classification using epigenomic data.

Research	Country	Target Disease	Data Type	Epigenomic Data Source	Validation Scheme	Predictive Model	Evaluation Metrics					
							AUC	Sensi-Tivity	Speci-Ficity	ACCU-RACY	Preci-Sion	F1-Score
Al Mamun et al. (2019) [32]	USA	8 types of cancer	long ncRNA	UCSC xena (TCGA)	–	MLP	–	0.929	–	0.937	0.932	0.939
						LSTM	–	0.952	–	0.956	0.951	0.951
						CNN	–	0.976	–	0.978	0.977	0.976
						DAE	–	0.959	–	0.964	0.961	0.960
Albaradei et al. (2019) [33]	Kingdom of Saudi Arabia	Colorectal cancer	DNAm	300 samples (TCGA)	15% of the dataset	CNN	–	0.967	0.958	0.962	0.904	0.947
Batbaatar et al. (2020) [34]	South Korea	12 types of cancer	DNAm	2728 samples (TCGA)	–	An incremental feature selection + a scholar network	–	–	–	0.932	–	–
Laplante et al. (2020) [35]	Canada	27 types of cancer	miRNA stem-loops	8573 cases (TCGA)	15% of the dataset	ANN	–	0.969	–	0.969	0.969	0.969
Levy et al. (2020) [36]	USA	32 types of cancer	DNAm	8891 samples (TCGA)	20% of the dataset	VAE + MLP	–	0.970	–	0.970	0.970	0.970
Smolander et al. (2019) [37]	Finland	Lung cancer	ncRNA	62 cases and 62 controls (GSE40419–GEO)	10-fold CV on the whole dataset	DBN	0.968	1.00	0.936	0.968	–	–
Titus et al. (2018) [38]	USA	Breast cancer	DNAm	86 normal-adjacent samples (TCGA)	Training/validation = 90/10	VAE + Logistic regression classifiers	–	–	–	0.961	–	–
				86 basal-like samples (TCGA)			–	–	–	0.944	–	–
				31 Her2 samples (TCGA)			–	–	–	0.961	–	–
				285 Luminal A samples (TCGA)			–	–	–	0.695	–	–
				124 Luminal B samples (TCGA)			–	–	–	0.843	–	–
Wang et al. (2019) [39]	China	Lung cancer	DNAm	507 LUAD samples (TCGA)	Training/validation = 90/10	VAE + Logistic regression classifiers	–	0.990	–	–	0.920	0.960
				412 LUSC samples (TCGA)			–	0.960	–	–	0.990	0.970
Zheng et al. (2020) [40]	USA	18 types of cancer	DNAm	7339 samples (TCGA)	10-fold CV on training set (60% of the dataset)	MLP	–	0.926	0.997	–	0.950	–

**Table 4:** Comparison of DL models for treatment response prediction using epigenomic data.

Research	Country	Target Disease/Drug	Data Type	Epigenomic Data Source	Validation Scheme	DL Model	Evaluation Metrics					
							AUC	Sensi-Tivity	Speci-Ficity	ACCU-RACY	Preci-Sion	F1-Score
Chang et al. (2020) [41]	Taiwan	Hepatitis B	RNA-seq	GSE101575 (GEO)	10-fold CV on the whole dataset	FC neural network	0.990	–	–	0.926	–	–
Morilla et al. (2018) [42]	France	Ulcerative colitis	miRNA	47 samples	Leave-one-out and K-fold CV on 29 samples	DNN	–	–	–	–	–	–
		Steroids					0.910	–	–	0.930	–	–
		Infliximab					0.820	–	–	0.840	–	–
		Cyclosporine					0.790	–	–	0.800	–	–

miRNA, microRNA; RNA-seq, RNA sequencing; GEO, Gene Expression Omnibus; CV, cross-validation; DNN, deep neural network; FC, fully-connected; AUC, area under the receiver operator characteristics curve; –, not available.

**Table 5:** Comparison of epigenomic data sources used for training predictive models for translational epigenomics.

Characteristics	Common Public Databases		Private Dataset
	The Cancer Genome Atlas	Gene Expression Omnibus	
Target disease	Cancer only	Various diseases	Various diseases
Data type	Clinical, gene copy number, DNA, imaging, methylation, microsatellite instability, microRNA, messenger RNA expression, protein expression	Gene expression, non-coding RNA, chromatin immunoprecipitation, DNA methylation, real-time PCR, genome variation profiling, single nucleotide polymorphisms arrays, serial analysis of gene expression, protein array	Depends on study objective
Data format	Raw, normalized, integrated data	Both raw and processed data submitted by the researchers	Raw data
Data source	Specific studies		Samples collected directly from patients

**Table 6:** Comparison of libraries used for training a DL model.

Library	Brief Description	Creator	Programming Language	Operating System	Links (accessed on 27 October 2021)
Deeplearning4J	Supports all the needs of the based DL application	Skymind	Python, Java, Scala, C++, C, CUDA	Linux, Win, OSX, Android	deeplearning4j.org
Keras	Focuses on enabling fast experimentation	Franois Chollet	Python, R, CUDA	Linux, Win, OSX	keras.io cran.r-project.org/web/packages/keras/
H_2_O	The scalable open-source machine learning platform that offers parallelized implementation of many supervised and unsupervised learning algorithms	Erin LeDell et al.	R, Java	Win, OSX, Ubuntu	cran.r-project.org/web/ packages/h2o
PyTorch	An optimized tensor library for DL using graphics processing units and central processing units	Facebook	Python, CUDA, C++	Linux, Win, OSX	pytorch.org
TensorFlow	Has a comprehensive and flexible suite of tools	Google	Python, C++, GO, Java, R, CUDA	Linux, Win, OSX, Android	tensorflow.org tensorflow.rstudio.com
Scikit-learn	Provides many supervised and unsupervised learning algorithms via a consistent interface	David Cournapeau et al.	Python, C, C++, Cython	Linux, Win, OSX	scikit-learn.org

DL, deep learning.

**Table 7:** Strengths and limitations of common evaluation metrics for classification performance of a DL model.

Evaluation Metric	Definition	Strength	Limitation
Accuracy	Fraction of correctly classified instances in the test set. A complement to the error-rate that measures fraction of the instances from the test set that are misclassified by the learning algorithm.	Summarize the overall performance.	Not relevant when either the performance on different classes is of varying importance or the distribution of instances in the different classes of the test data is skewed.
True positive rate (Sensitivity or recall)	Proportion of actual positives which are correctly identified.	Ameliorates the effect of class imbalance arising in the accuracy or error-rate measurements thereby skewing these estimates.	In the case of a multiclass classification problem, this would lead to as many metrics as there are classes, making it difficult to interpret.
True negative rate (Specificity)	Proportion of actual negatives which are correctly identified.		
Positive predictive value (Precision)	Proportion of relevant examples (true positives) among all of the examples which were predicted to belong in a certain class.	Gives an insight into how reliable the class-wise predictions of a classifier is.	Might not provide enough information for a concrete judgment call on the superiority of the classifier in one case or the other.
F-score	An even weighted harmonic mean of precision and recall. The most commonly used metric is F1-score that weights the recall and precision of the classifier evenly.	Leaves out the true-negative performance of the classifier.	Ignores true negatives and thus is misleading for unbalanced classes. Appropriate weights for combining the precision and recall are generally not known.
Receiver operator characteristics (ROC) curve	A plot which takes true positive rate as the vertical axis and false positive rate as the horizontal axis.	Visualizes the performance of classifiers over their operating ranges.	Unable to quantify the comparative analysis that can facilitate decision making with regard to the suitability or preference of one classifier over others in the form of an objective scalar metric.
Area under the ROC curve (AUC)	Entire two dimensional area underneath the entire ROC curve.	Provides an aggregate measure of performance across all possible classification thresholds.	Loses significant information about the behavior of the learning algorithm over the entire operating range.

## Data Availability

Not applicable.
